# Effect of Acting Experience on Emotion Expression and Recognition in Voice: Non-Actors Provide Better Stimuli than Expected

**DOI:** 10.1007/s10919-015-0209-5

**Published:** 2015-02-26

**Authors:** Rebecca Jürgens, Annika Grass, Matthis Drolet, Julia Fischer

**Affiliations:** 1 Cognitive Ethology Laboratory, German Primate Center, Kellnerweg 4, 37077 Göttingen, Germany; 2 Courant Research Centre “Evolution of Social Behaviour”, University of Göttingen, Göttingen, Germany; 3 Present Address: Courant Research Centre “Text Structures”, University of Göttingen, Göttingen, Germany

**Keywords:** Acoustics, Actors, Emotion, Play-acting, Vocal expressions

## Abstract

Both in the performative arts and in emotion research, professional actors are assumed to be capable of delivering emotions comparable to spontaneous emotional expressions. This study examines the effects of acting training on vocal emotion depiction and recognition. We predicted that professional actors express emotions in a more realistic fashion than non-professional actors. However, professional acting training may lead to a particular speech pattern; this might account for vocal expressions by actors that are less comparable to authentic samples than the ones by non-professional actors. We compared 80 emotional speech tokens from radio interviews with 80 re-enactments by professional and inexperienced actors, respectively. We analyzed recognition accuracies for emotion and authenticity ratings and compared the acoustic structure of the speech tokens. Both play-acted conditions yielded similar recognition accuracies and possessed more variable pitch contours than the spontaneous recordings. However, professional actors exhibited signs of different articulation patterns compared to non-trained speakers. Our results indicate that for emotion research, emotional expressions by professional actors are not better suited than those from non-actors.

Acting is not only an essential part of human performative culture, but also of everyday social life, since emotion expressions in natural settings are frequently play-acted due to social requirements (Goffman 1959; Gross 1998; Hochschild 1979; Kappas 2013). At the same time, actors’ portrayals can be strongly influenced by subjective feelings, especially when produced via techniques based on emotional imagination or memory (Gosselin et al. 1995; Scherer and Bänziger 2010). Therefore, it has been argued that genuine expressions of emotions and play-acted ones are difficult, if not impossible, to distinguish (Scherer and Bänziger 2010). Other authors have criticized clearly staged expressions as being stereotypical, exaggerated and more intense than spontaneously occurring expressions (Barrett 2011; Batliner et al. 2000; Douglas-Cowie et al. 2003). However, only a handful of studies have directly compared authentic expressions and actors’ portrayals (Aubergé et al. 2004; Drolet et al. 2012; Greasley et al. 2000; Laukka et al. 2012; Williams and Stevens 1972). In most of these studies, play-acted expressions were found to be more intense or more stereotypical (Laukka et al. 2012; Wilting et al. 2006). Yet, some more recent studies failed to detect such a pattern (Drolet et al. 2012; Jürgens et al. 2013; Scherer 2013).

In a set of earlier studies, we compared vocal expressions of emotions taken from natural, non-staged situations recorded by a local radio station with their re-enactments by professional actors. In the course of the paper we will use the terms “authentic,” “play-acted,” and “realistic” according to the following definitions. “Authentic” is used for stimuli that are recorded in spontaneous non-staged, daily life situations, reflecting the expressions we use in our day-to-day emotion communication. The term does not reflect the physiological (affective) state or inner feelings of the encoder. “Play-acted” stimuli are recorded under the instruction, to transmit specific emotional information using a given wording, without an intrinsic motivation of the encoder. “Realistic” is used for play-acted stimuli that are perceived as authentic, that is believed to be spontaneous. Our results showed that listeners were poor at identifying the encoding condition (that is whether the stimuli were authentic or play-acted). Furthermore, in contrast to the prediction that they are more stereotypical, play-acted expressions were not generally recognized more accurately (Drolet et al. 2012; Jürgens et al. 2013). Instead, we found a significant interaction between emotion category and encoding condition with anger being recognized more frequently when play-acted, while sadness was recognized more frequently when authentic. This effect has been replicated across different cultures (Jürgens et al. 2013). An imaging study comparing brain activation via BOLD response (blood oxygenation level dependent, measured by functional magnetic resonance imaging) of the authentic and play-acted stimuli showed that listening to the authentic but not to the play-acted stimuli activates the Theory of Mind network (ToM) (Drolet et al. 2012). The encoding condition of emotional stimuli thus interacts with neural processing, indicating its importance on human response behavior.

A comparison of the acoustic structure revealed differences in articulation and a more variable pitch contour for play-acted stimuli, showing that measurable differences in the stimulus material between play-acting and authentic encoding condition exist (Jürgens et al. 2011). As we compared not only acting to non-acting but professional actors’ voices to normal people’s voices and speaking style, our results raised the question whether the effects referred to acting in general, or to the elaborated articulation of professional actors.

Professional actors may produce emotional expressions that are more realistic than expressions by lay people (hereafter “non-actors”), due to their acting training (hypothesized by Krahmer and Swerts 2008; Scherer and Bänziger 2010). Specific acting styles include own feelings as part of the actors’ performance; these methods require extensive training and are supposed to increase realism, precisely because they rely on inner affective states, thereby emphasizing the advantage of using actors for creating emotional stimuli that resemble expressions in spontaneous situations (Enos and Hirschberg 2006; Gosselin et al. 1995; Scherer and Bänziger 2010).

Actors, however, need to transmit their emotional expression to the back row of the theater, which might lead to overexpression (Kracauer 2005) and their speech training may influence phonation and articulation in order to produce loud, intelligible, and persisting speech (Master et al. 2008; Nawka et al. 1997; Roy et al. 2000). Thus, professional actors may not necessarily produce more realistic emotional expressions compared to non-actors (see Krahmer and Swerts 2008). Spackman et al. (2009) tested the influence of acting training on emotional expressions in voice comparing eight drama students with an inexperienced control group. An acoustic comparison revealed interaction effects between encoding conditions and the acoustic structure for single emotions. From the perspective of the listener, anger and fear stimuli produced by actors were recognized more accurately than those by laymen, but the reverse was true for happiness and sadness. Krahmer and Swerts (2008) induced positive and negative affective states in their participants via the Velten induction method (Velten 1968) and compared the resulting facial expressions with portrayals by experienced theatre actors and non-actors. Contrary to their prediction, facial expressions by actors were perceived as the most intense. These studies indicate that professional actors may not be more suited to producing emotional expression than non-actors, at least when resemblance to spontaneous expressions is the goal.

Our study aims to deepen the understanding of training effects on vocal expressions of emotions and to put them in relation to expressions produced in spontaneous situations. With this approach, we aim to advance the discussion about what differences concerning the effect of authenticity (Drolet et al. 2012; Jürgens et al. 2011, 2013) are due to acting per se and which might be due to the actors’ way of speaking.

We formulated two opposing hypotheses: (1) If professional actors are more suited to producing realistic emotional expression through their acting training, we would predict that the acoustic structure of non-actors’ speech tokens deviate more from the authentic expressions than the actors’ portrayals. In this case, we would predict that portrayals by non-actors were more stereotypical and exaggerated and, thus, were more easily recognized as being play-acted. Recognition accuracies for the emotion categories would be the highest for the non-actors’ expressions. (2) If however, professional acting and speech training leads to different speech patterns, we would predict that expressions by professional actors differ from the other conditions, both in their acoustic structure and in their perception, while the differences between non-actors and authentic emotion expressions were negligible. Recognition accuracies both for authenticity recognition and emotion recognition would in this case be the highest for actors. Based on earlier research (Jürgens et al. 2011), we made clear predictions for the acoustic parameters. In the past decades acoustic parameters have been described that differentiate the expressions of different emotion categories (Hammerschmidt and Jürgens 2007; Juslin and Laukka 2001; Scherer 1986). These parameters mirror the phonation (sound production) and the articulation process (modulation of sound via nasal and oral cavities) respectively. Highly aroused emotions such as anger, are spoken faster, less monotonously, more loudly, with more energy in the higher frequencies, more noise in the signal and in a higher fundamental frequency (pitch); while low aroused emotions such as sadness are spoken slower, monotonously, quietly, with more energy in the lower frequencies, with less noise, and in a lower fundamental frequency. Speed of speech, speech melody, fundamental frequency, harmonic-to-noise-ratio, and peak frequency are thus parameters that distinguish emotional speech and that are related to arousal differences in general (Juslin and Laukka 2001; Scherer 1986). In the previous study on the extensive acoustic analysis of the authentic and professionally acted expressions (Jürgens et al. 2011), none of these parameters differed systematically between authentic and actors’ speech tokens, with the exception of peak frequency, which was found to be slightly lower in play-acted expressions and the more variable speech melody in acted portrayals. The most pronounced differences between actors and authentic speech tokens were the broader bandwidths of the first formants, the more dominant fundamental frequencies, both of which are not affected by emotion, and the more variable pitch contour (speech melody) in actors’ speech (Jürgens et al. 2011). If non-actors’ portrayals were more exaggerated (and thus more aroused) than actors’ expressions, we would predict higher values for the arousal related parameters (fundamental frequency, speed of speech, peak frequency, harmonic-to-noise ratio, energy distribution, and pitch contour) in the non-actors condition. Additionally, the bandwidths of the first formant and the more dominant fundamental frequency should be even more pronounced. However, if the articulation and modulation differences are something related to the actors’ voice, we predict negligible differences between non-actors and authentic speech in these acoustic structures.

## Method

### Stimuli

#### Authentic

The authentic speech recordings were selected from the database of a German radio station and were taken from interviews made while the individuals were talking about an emotionally charged on-going situation or describing their emotional state while recollecting a past event. 80 recordings were selected that had a good recording quality and a low amount of background noise. The selected recordings contained interviews in which the individuals expressed anger, fear, sadness, or happiness (specified via situation context and verbal content of the recordings). The radio recordings were then converted into wav files (sampling rate of 44.1 kHz). From these interviews, short segments up to 5.5 s in length were cut and consisted of neutral verbal content that does not indicate any specific emotion. Neutral content was rated prior to the study by an additional set of 64 naïve participants. Only these short segments, which ranged from three words to half-sentences, were used for the study, such as “up to the crossbar” [German original: “bis zum Fensterkreuz”], “twice in a row and such” [“zweimal hintereinander und so”], and “read it again” [“lesen Sie es noch mal vor”]. The 80 speech segments were spoken by 78 speakers and consisted of 22 anger, 18 fear, 20 joy, and 20 sadness stimuli (half spoken by female speakers). The emotional content of the recordings (whether we classified the interview to the anger, joy, fear, or sadness condition) was determined via context analysis by a post-doctoral member of our research group. Recordings in which speakers were talking about a loss were categorized as sadness, while situations regarding winning and celebration were categorized as happiness. Recordings in which people reported or lived through a threatening event were grouped as fear and the ones in which people verbally attacked someone were grouped as anger. The selected recordings represented a broad variety of emotion situations and emotion intensities. We could neither exclude the possibility of mixed emotions by the sender nor could we control their actual physiological affective state. However, our focus was on the natural communication of emotion that is seldom clearly distinct and controlled. The recording instructions of the actors and non-actors were adjusted to allow for comparable mixed expressions. Examples of the stimuli and of the context situations are found in the “Appendix”.

#### Play-Acted Expressions

Professionally play-acted stimuli (hereafter stimuli produced by “actors”) were produced by 21 male and 21 female actors (*M* age = 31 years, SD = 7.9, age range 21–48 years), 30 of them were professional actors who mainly worked on stage, 11 were acting students at the end of their education and one was a professional singer with acting experience. All of the actors had taken part in professional acting training. They were asked to enact one to three of the authentic recordings. Most of them (33 of 42) reproduced two original recordings with the same intended emotion that is two times anger, or two times joy, respectively. The actors used an information sheet (indicating the gender of the speaker, a situational description, and a transcription of the spoken text, including the respective text segment later used for the study) and were told to express the text in their own way. The respective emotion was mentioned in the situational description (e.g., “…She said full of joy…, …an inhabitant reports her fears…, she reports her pain and her sadness…, he got terribly agitated…”). This allows mixed emotions and different intensities expressed by the actors to mirror the recording condition of the authentic expressions. The actors were instructed not to speak in their stage voice, to imagine the situation and to feel into it. The short segment that was later used for the study was not known to the actors. Actors were allowed to express the text as often as they wanted and could select the expression they considered to be most successful. The recordings were made with a Marantz Professional Portable Solid State Recorder (Marantz, Kenagawa, Japan) with a sample rate of 44.1 kHz, a sampling depth of 16 bit and a Sennheiser directional microphone (Sennheiser, Wedemark, Germany, K6 power module and ME64 recording head).

Non-professionally play-acted recordings (“non-actors”) were recorded similarly. Twenty women and 19 men (*M* age = 45 years, SD = 14.8, age range 21–67 years) were recruited via postings at a university notice board and by recruitment in the second authors’ circle of acquaintances. The sample of non-actors consists of students, teachers, and normal employees. The non-professional actors were thus older on average than the professional actors; however, age classes could not be determined for the authentic speakers. Ten of the speakers indicated experiences in amateur theatre groups (like school theatres), but none of them received professional acting training. Recordings were made using the same procedure and the same transcriptions as for the actors and were made with a Field Memory Recorder (Fostex, Tokyo, Japan, FR-2LE) and a Sennheiser directional microphone (Sennheiser, Wedemark, Germany, K6 power module and ME64 recording head) with a sampling frequency of 44.1 kHz and a 16 bit sampling depth. To reduce category effects between authentic and play-acted stimuli, both the professional and the non-professional re-enactments were partly recorded outside with varying background noise, as the radio recordings also varied in their background noise.

For all three conditions, the recordings were edited with AvisoftSASLab Pro Version 5.1 (AvisoftBioacustics, Berlin, Germany) to cut the short segments used for the study out of the longer interviews. The final stimulus set consisted of 240 short text segments (80 authentic, 80 professional play-acted, and 80 non-professional play-acted) with non-emotional text content (e.g., “up to the crossbar”) flanked by 0.5 s silences. The mean duration of all stimuli was 1.87 s (*SD* = 1.29, range 0.327–8.03 s). Duration did not vary between the encoding conditions (*M* authentic = 1.89 s, *M* actors = 1.95 s, *M* non-actors = 1.79 s, linear mixed model (LMM) comparison *χ* ^*2*^ = .114, *df* = 2, *p* = .946).

### Rating Experiment

#### Design

The 240 stimuli were divided into four sets of 60 stimuli made up of 20 “authentic” stimuli, 20 “actors,” and 20 “non-actors” expressions so that subjects were not confronted with the same sentence spoken by the authentic speaker, the actor, and the non-actor, in order to avoid a direct comparison of sentences. The 60 stimuli of one set were selected in such a way that there was neither a repetition of one specific stimulus (e.g., an authentic stimulus and the same stimulus re-enacted by an actor) nor a repetition of one speaker in one set. Each participant listened thus to only one-fourth of the whole stimulus material.

The rating experiment was performed using the program NBS Presentation (Neurobehavioral Systems, Inc., Albany, California). Participants had to evaluate each of the 60 stimuli in regards to the specific vocal expression of emotion and to authenticity. During the experiment they either had to rate whether the stimulus represents “anger” [German original: Wut], “fear” [Angst], “sadness” [Trauer], or “joy” [Freude] (emotion recognition), and whether it is “play-acted” [gespielt], or “authentic” [echt] (authenticity recognition). The sets were pseudo-randomized to avoid serial repetition of trial order (order of the emotion and authenticity judgment task) more often than two times, of encoding condition (“authentic,” “non-actors,” or “actors”) more often than two times, and of intended emotion more often than three times. This was done to reduce any systematic or pattern-related effects. In addition, the order of the four possible emotion-responses (“joy,” “anger,” “fear,” and “sadness”) and the two possible authenticity-responses (“authentic” and “play-acted”) was counterbalanced per participants to avoid enhancement of a specific response by preferential effects for a specific response button.

#### Participants and Experimental Procedure

Participants for the rating experiment were recruited in the Cafeteria of the Georg-August-University of Göttingen and at the German Primate Center, Göttingen, Germany. They were all native German speakers. Two-hundred and twenty-eight subjects participated (69 female and 59 male) in the rating experiment. The subjects were students (*N* = 99) or scientific assistants (*N* = 29). Every single stimulus was thus rated by 32 subjects. Eighty of the subjects were between 18 and 24 years of age, 36 between 25 and 29 years, seven between 30 and 34 years and five subjects 35 years or older.

The stimuli were played back with a laptop (Toshiba Satellite M70-187 with a Realtek AC97 Soundcard) via NBS Presentation. Subjects heard the stimuli via earphones (Sennheiser HD 448 and HD 280 pro). Before the experiment started, subjects read a description about their task and the experimental procedure. All remaining questions were answered before the experiment started, after which there was no further interaction between participant and experimenter and the trials were played back automatically by Presentation as defined in the script.

#### Ethics

The study was approved by the ethics committee of the Georg-Elias-Müller-Institute of Psychology (University Göttingen). Professional and non-professional actors consented to the use of their shortened recordings in our rating experiment and to the anonymous acoustic analysis. Professional actors were paid 20 Euros for their participation. The non-professional actors and the participants of the rating experiment received candy bars for their participation. For the rating study, we did not obtain informed consent as data was collected and analyzed anonymously.

### Acoustic Analysis

The acoustic analysis was conducted on two levels—on single vowels (Level 1) and on the short speech sequence (Level 2). Level 1: Vowels (a, e, i) were cut out of the speech tokens to obtain comparable units. For these vowels, we calculated the mean fundamental frequency (F0), the harmonic-to-noise-ratio, the frequency with the highest amplitude (peak frequency), the bandwidth of the first formant (hereafter “first formant”) and the amplitude ratio between third frequency band and F0 (hereafter “amplitude ratio”). All measurements were conducted using the spectrogram analysis software *LMA* (Lautmusteranalyse, developed by K. Hammerschmidt), except the first formant that was analyzed using *Praat* (Boersma and Weenink 2009). We used this set of parameters, as they mirror the phonation process (F0, harmonic-to-noise ratio) and the articulation (peak frequency, first formant, amplitude ratio). Furthermore, they are independent of each other, known to be affected by emotions (F0, harmonic-to-noise ratio, and peak frequency) and were already used in the comparison between the professional actors and the authentic recordings (see Jürgens et al. 2011).

Level 2: We measured the speech tempo and the variability of the F0-contour for the entire speech tokens that were used in the rating experiment. To determine the speech tempo, we calculated the speech rate (syllables/sec including pauses) and the articulation rate (syllables/sec excluding pauses). The variability of the F0-contour was measured via the standard deviation of the F0 for each speech token. All measurements were done manually using AvisoftSASLab. For a detailed description of the acoustic analysis see Jürgens et al. (2011).

### Statistical Analysis

#### Recognition of Encoding Condition

The statistical analysis was done using R (R Developmental Core Team 2012). Pure recognition rates reflect the behavior of the listener, but do not mirror the listeners’ actual ability to distinguish the categories. High recognition rates in one condition might simply be caused by the participant’s bias to only or preferentially choose the respective response category. Therefore, we calculated unbiased hit rates according to Wagner (1993). Unbiased hit rates reflect the probability of one participant that a stimulus is correctly recognized and that a response is correctly given, thus incorporating individual biases in response behavior. We tested the effect of *emotion* (“anger,” “fear,” “sadness,” or “joy”), *encoding condition* (“authentic,” “actors,” or “non-actors”) and their interaction on the recognition of encoding condition establishing a LMM (lmer function of the lme4 R package Bates et al. 2011). As “actors” and “non-actors” provided two to three stimuli to the dataset, we had to deal with the dependency among our data. For the rating experiment, we divided the data set into four sets, so that participants rated only one speech token of every actor. We then included *participant*-*ID* and *stimulus block* (1–4, representing the set, in which the stimulus was presented) as random effects into the statistical model to account for the influence of these variables. *Participant gender* was added as a fixed factor, while speakers’ gender was not, as the calculation of individually unbiased hit rates incorporated every stimulus presented to one participant. Unbiased hit rates are proportions and were thus arcsine transformed prior to the analysis. The full model was compared to the null model (only including intercept and random factors) using a likelihood ratio test (function ANOVA with the test argument “Chisq”), to establish the significance of the full model. The interaction effect of both categorical predictors was also tested using a likelihood ratio test. Afterwards, we conducted twelve post–hoc comparisons for all emotions between the three encoding conditions using the function glht (from the package multcomp Hothorn et al. 2008). *p* values were adjusted using a Bonferroni correction.

Following the suggestion by Wagner, we calculated for every participant the chance probability that a stimulus is recognized correctly and compared the unbiased hit rates to the chance levels. The statistical model was built with hit rates as the response variable, *type of hit rate* (unbiased or chance), *encoding condition* and *emotion* as fixed factors, as well as *participant*-*ID* as a random effect. The full model was compared to the null model; post hoc tests for *type of hit rate* (chance or unbiased hit rate) regarding every condition were done using the glht function, *p* values were Bonferroni corrected.

#### Emotion Recognition

Similar to the recognition of encoding condition, we calculated unbiased hit rates as well as the respective chance probabilities for the emotion recognition. The analysis followed the procedure mentioned above for the recognition of encoding condition.

#### Acoustic Structure of Vowels

Altogether we included 1176 vowels into the analysis, divided into 446 by authentic speakers, 346 by actors, and 384 by non-actors. We analyzed the acoustic parameters separately for vowels a, e, and i as a previous study revealed interaction effects between encoding condition and vowels (Jürgens et al. 2011). The effects on the acoustic parameters *F0, harmonic*-*to*-*noise ratio, peak frequency, amplitude ratio,* and *bandwidth of the first formant (*hence *first formant)* were tested using LMMs. *Emotion, encoding condition, speaker’s gender,* and the interaction between *emotion* and *encoding condition* were entered as fixed factors. As random effects, we included *speaker*-*ID* and *stimulus*-*text.* Normal distribution and homogeneity of residuals were tested by inspecting Quartile-Quartile-Plots (QQ-plots) and residual plots. For the following acoustic parameters (a, e, and i) a deviation of assumptions was found and they were thus log transformed: the amplitude ratio, the peak frequency, and the first formant. We then compared the full models to the null models (function ANOVA test argument “Chisq”) to establish significance of the models. We tested for interaction effects by comparing the model including the interaction with the model excluding the interaction and used the reduced model when appropriate (function anova test argument “Chisq”). Main effects for fixed factors were also tested by model comparisons. We treated the acoustic parameters separately and adjusted the *p* values with a Bonferroni correction for multiple testing within the three vowels. Finally, we conducted a post hoc analysis for the acoustic parameters that were affected by encoding condition (function glht with Bonferroni adjustment for all possible comparisons).

#### Speed of Speech

We tested the influence of *emotion*, *encoding condition* and their interaction on both *speech rate* and *articulation rate* by using LMMs (lmer from the package lme4). *Stimulus text* and *speaker*-*ID* was entered as a random effect. The assumptions of normal distribution and homogeneous residuals were tested by inspecting the QQ-plots and the residual plots. We then compared the full model to the null model using the likelihood ratio test of the function ANOVA (test function “Chiq”).

#### Pitch Variability

We tested the influence of *encoding condition* on the *F0*-*standard deviation (F0*-*SD)* by using LMMs (lmer from the package lme4). We included *encoding condition* as predictor and *stimulus text and speaker*-*ID* as random effect into the model. To obtain a normal distribution of the data, *F0*-*SD* was log transformed. The assumptions of normal distribution and homogeneous residuals were tested by inspecting the QQ-plots and the residual plots. The variability of pitch contour depends strongly on the type of sentence (that is exclamatory or interrogative sentence, beginning, or end of a sentence) or on stimuli length. Therefore, different sentences cannot be compared without restrictions. For the encoding condition, we could compare the same sentence across different modalities (as every sentence was present in every condition), which could not be done for the emotion effects. Hence, emotion was not regarded as a predictor for this analysis. We compared the full model to the null model (test ANOVA, test function “Chiq”) to look for an effect of *encoding condition* on *F0*-*SD* and used the function glht (with Bonferroni adjustment) for post hoc testing.

## Results

### Rating Experiment

#### Recognition of Encoding Condition

**Fig. 1:**
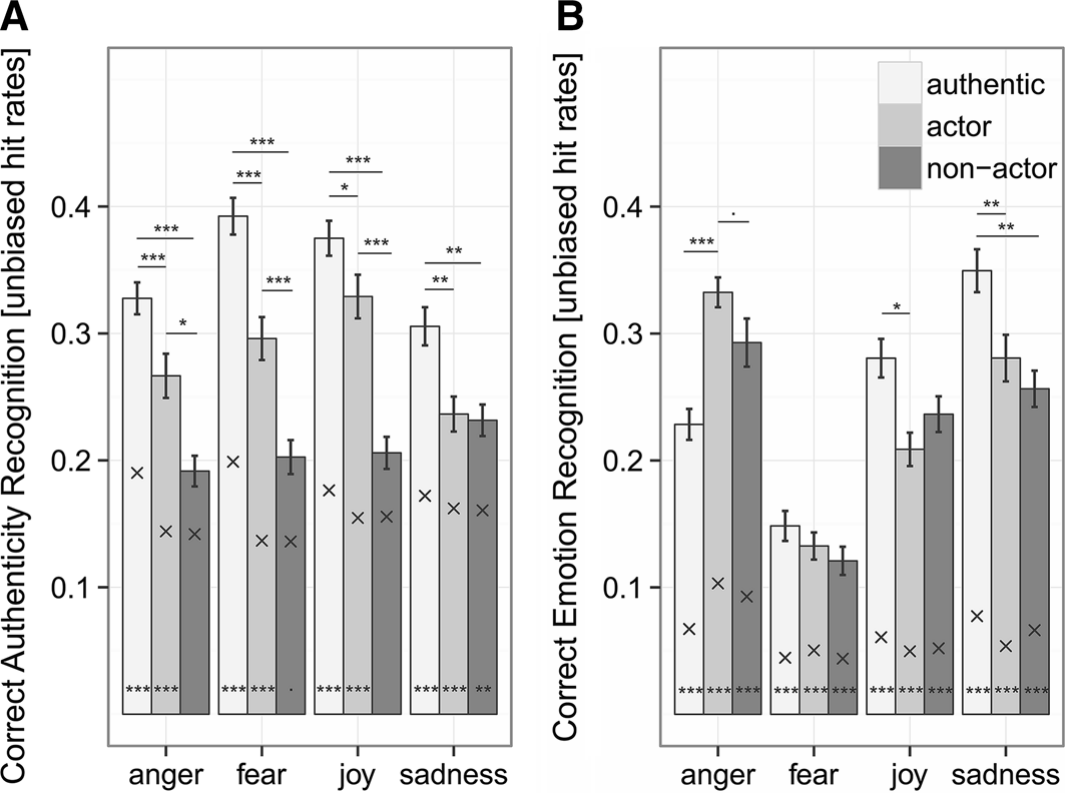
Recognition of encoding condition (**a**) and emotion recognition (**b**) across all conditions. **a** Unbiased hit rates for recognition of encoding condition (responding “authentic” when the stimulus is authentic, “play-acted” when stimulus is either from actors or non-actors), given as mean values ± *SEM*. **b** Unbiased hit rates for the emotion classification (mean values ± *SEM*). X marks the mean chance levels per condition. *Asterisks* refer to the significance level. < .1; *p < .05; **p < .01, ***p < .001. *Asterisks* at the *bottom* of the *bars* indicate differences between unbiased hit rates and individual chance level

Encoding condition was correctly classified in 59.8 % of all cases. The authentic recordings were recognized correctly as being authentic in 72.7 % of all cases, while the actors’ recordings were recognized as being play-acted in 57.5 %, and the non-actors’ recordings in 49.1 % of all cases. The comparison to the null model established an overall effect of the predictors on unbiased hit rates (*χ* ^*2*^ = 314.31, *df* = 12, *p* < .001). Additionally, an interaction effect between emotion and encoding condition was found (interaction: *χ* ^*2*^ = 46.684, *df* = 6, *p* < .001, Fig. 1). Post-hoc tests revealed that authentic speech tokens were recognized as such with the highest accuracy, regardless of the emotion category. Actors’ expressions were recognized more accurately than the non-actors’ expression, but only for anger, fear, and joy stimuli (Table 1; Fig. 1). Participant gender (*χ* ^*2*^ = 0.2156, *df* = 1, *p* = .642) had no effect on the recognition of the encoding condition. The recognition rates were generally quite low, indicating a poor ability to judge encoding condition. In fact, recordings by non-actors of anger, fear, and joy were not recognized above chance level (post-hoc comparison for anger: *z* = 2.071, *p* = .460, fear: *z* = 2.861, *p* = .0505, and joy: *z* = 1.778, *p* = .904). All other conditions were recognized above chance (all *z* > 3.660, *p* < .003). In sum, the *encoding condition* was only poorly recognized by the participants, but nevertheless differed between encoding conditions. Although the authentic stimuli were recognized at a higher rate than the acted stimuli, both acting conditions were often misjudged as being authentic as well. The portrayals by non-actors were significantly more frequently misjudged as being authentic than the expressions by professional actors.

**Table 1:** Post-hoc comparisons for recognition of encoding condition and emotion (unbiased hit rates)

Type	Emotion	Encoding conditions		Estimate^a^	*SE*	z value	*p* ^b^
Encoding condition	Anger	Authentic	Actors	0.00848	0.002049	4.138	<.001***
		Authentic	Non-actors	0.01520	0.002049	7.418	<.001***
		Actors	Non-actors	0.00672	0.002049	3.279	.013*
	Fear	Authentic	Actors	0.01057	0.002049	5.159	<.001***
		Authentic	Non-actors	0.02029	0.002049	9.901	<.001***
		Actors	Non-actors	0.00972	0.002049	4.743	<.001***
	Joy	Authentic	Actors	0.00545	0.002049	2.661	.094
		Authentic	Non-actors	0.01795	0.002049	8.76	<.001***
		actors	Non-actors	0.01250	0.002049	6.099	<.001***
	Sadness	Authentic	Actors	0.00800	0.002049	3.902	.001**
		Authentic	Non-actors	0.00772	0.002049	3.769	.002**
		Actors	Non-actors	−0.00027	0.002049	−0.133	1
Emotion	Anger	Authentic	Actors	−0.01111	0.002314	−4.8	<.001***
		Authentic	Non-actors	−0.00465	0.002314	−2.009	.535
		Actors	Non-actors	0.00646	0.002314	2.792	.063.
	Fear	Authentic	Actors	0.00116	0.002314	0.501	1
		Authentic	Non-actors	0.00405	0.002314	1.749	.964
		Actors	Non-actors	0.00289	0.002314	1.248	1
	Joy	Authentic	Actors	0.76010	0.002314	3.285	.012*
		Authentic	Non-actors	0.00464	0.002314	2.005	.539
		Actors	Non-actors	−0.00296	0.002314	−1.28	1
	Sadness	Authentic	Actors	0.00776	0.002314	3.353	.0096**
		Authentic	Non-actors	0.00886	0.002314	3.829	.0016**
		Actors	Non-actors	0.00110	0.002314	0.47	1

Asterisks mark the significance level at *p < .05; **p < .01; ***p < .001

^a^Based on arscine transformed data

^b^Adjusted *p* values (Bonferroni correction)

#### Recognition of Emotion

The different emotions were correctly recognized in 44.7 % of cases, with a recognition accuracy of 46 % for authentic stimuli, 45 % for actors’ recordings and 43 % for non-actors’ recordings. Regarding the recognition accuracy (unbiased hit rates), the full model, including *emotion*, *encoding condition* and their interaction, was significantly different from the null model (*χ* ^*2*^ = 251.55, *df* = 12, *p* < .001). Furthermore, we found a significant interaction between *emotion* and *encoding condition* (*χ* ^*2*^ = 30.565, *df* = 6, *p* < .001) (Fig. 1). Post-hoc-tests between “authentic,” “actors,” and “non-actors” for all four emotions revealed that professionally acted anger was recognized better than authentic anger, while the reverse was true for joy and sadness. Non-actors’ expressions showed a less accurate sadness recognition than the authentic speech tokens (Table 1). Overall, unbiased hit rates for expressions by professional actors were similar to the ones by non-actors, while both differed from the expressions in the “authentic” condition. No effect of participant gender on the emotion recognition was found (*χ* ^*2*^ = 0.2601, *df* = 1, *p* = .610). Unbiased hit rates were very low, but differed in every emotion condition from the individual chance level, even in the fear condition (post hoc comparison for every condition, unbiased hit rates—chance level: *z* > 4.94, *p* < .001). Fear was seldom recognized, but when participants gave the response “fear,” they were mostly correct.

### Acoustic Analysis

#### Acoustic Structure of Vowels

**Fig. 2:**
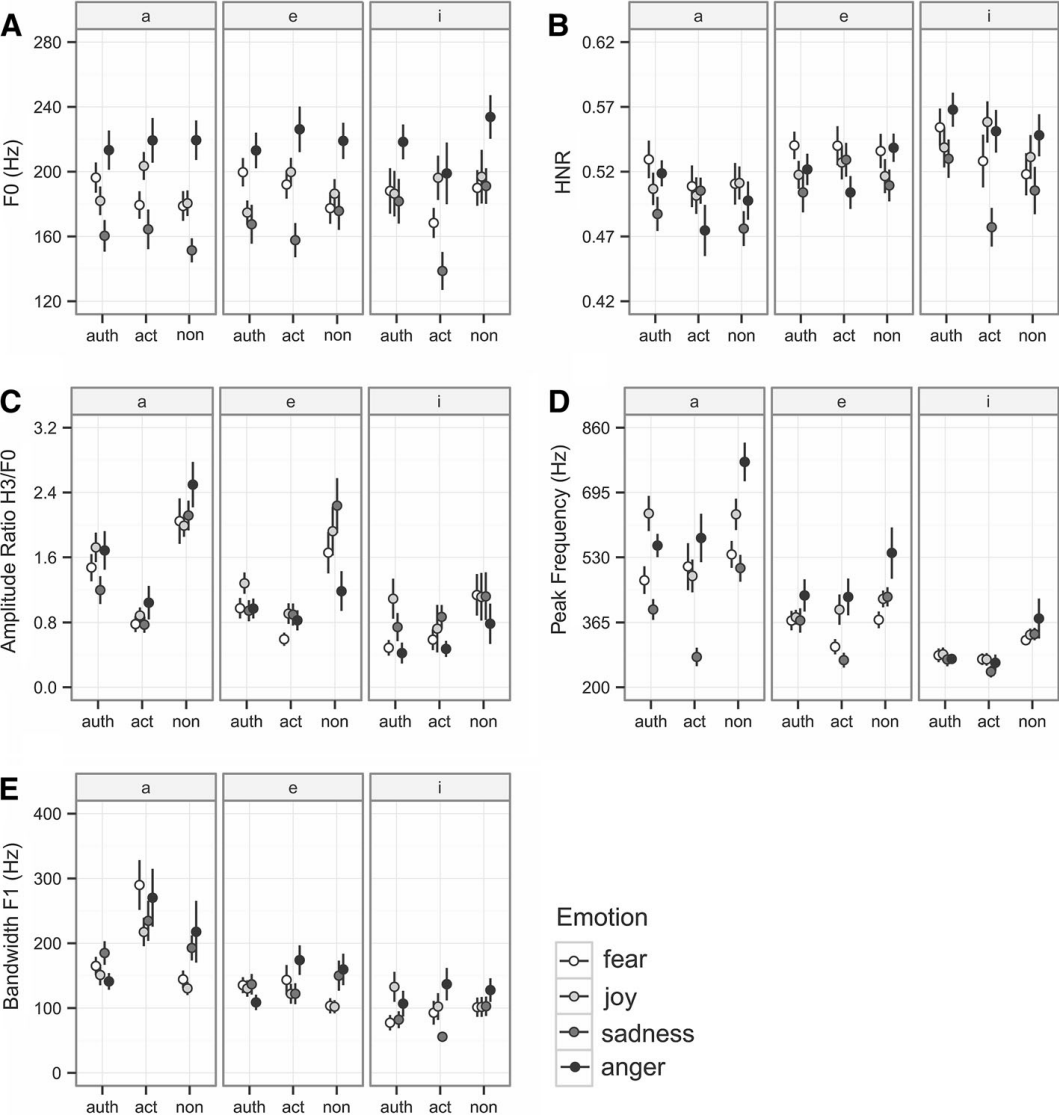
Selected acoustic parameters separated for emotion and encoding condition. Mean values are given for vowels a, e, i ± *SEM*

The variables in the analysis were affected by encoding condition, emotion, and gender (comparison of full models with null models; all *χ* ^*2*^ > 29.381, *df* = 12, *p* < .01), except for the first formant (vowels “e” and “i”) (Chi-statistics lower than *χ* ^*2*^ = 12.713, *df* = 12, *p* = 1). We found no interaction between *emotion* and *encoding condition* in any of the acoustic parameters (Chi-statistics lower than *χ* ^*2*^ = 7.65, *df* = 6, *p* = .7). Figure 2 shows the pattern of the acoustic variables in the different conditions. The acoustic profiles of all three conditions varied, with the biggest variation being between “non-actors” and “actors” speech tokens.

Notably, acoustic differences were putatively related to articulation. Specifically, amplitude ratio, peak frequency, and the bandwidth of the first formant (Table 2; Fig. 2) varied between “authentic,” “actors,” and “non-actors” recordings. The professional actors’ speech tokens differed most strongly from the other two encoding conditions. The authentic and the non-actors’ recordings also varied between each other, although they deviated in a similar way from the actors’ speech tokens. For instance, professional actors had a lower amplitude ratio (referring to a pronounced F0 and a less intense third frequency band) and wider bandwidths of the first formant compared to the “authentic” and the “non-actor” expressions; “authentic” and “non-actors” did not differ in their formant bandwidths, but “non-actors” vowels possessed higher amplitude ratios than the “authentic” ones (see Table 3 for the results of the post hoc analysis). To sum up, the acoustic structure varied between “authentic,” “actors,” or “non-actors” recordings, but encoding condition affected other variables than those affected by emotion (see below).

**Table 2:** Results of the linear mixed models on the acoustic structure of vowels

Parameter	Vowel	Emotion^a^		Encoding condition^a^		Gender^a^		
		$\upchi_{(3)}^{2}$	*p* ^b^	$\upchi_{(2)}^{2}$	*p* ^b^	$\upchi_{(1)}^{2}$	*p* ^b^	Estimates ± SE^c^
F0	a	15.34	.005**	0.42	1	43.56	<.001***	70.49 ± 9.72
	e	15.02	.005**	0.86	1	45.07	<.001***	69.72 ± 9.30
	i	14.48	.007**	3.41	.540	32.12	<.001***	70.18 ± 11.46
HNR	a	1.35	1	1.28	1	21.15	<.001***	0.046 ± 0.01
	e	3.32	1	0.19	1	22.99	<.001***	0.045 ± 0.009
	i	11.91	.023*	2.59	.822	41.78	<.001***	0.076 ± 0.010
Amplitude ratio	a	5.61	.397	52.07	<.001***	5.09	.072	−0.221 ± 0.099^d^
	e	1.74	1	26.15	<.001***	27.61	<.001***	−0.651 ± 0.117^d^
	i	11.65	.026*	3.79	.450	32.44	<.001***	−1.028 ± 0.164^d^
Peak frequency	a	21.42	<.001***	24.22	<.001***	0.76	1	0.067 ± 0.0774^d^
	e	8.13	.13	17.53	<.001***	6.99	.024*	0.169 ± 0.064^d^
	i	0.55	1	24.58	<.001***	6.50	.032*	0.121 ± 0.048^d^
First formant	a	4.66	.198	19.08	<.001***	5.00	.025*	0.190 ± 0.086^d^

Asterisks mark the significance level at *p < .05; **p < .01; ***p < .001

^a^Statistical values are obtained from the model comparison (full model to reduced model excluding the respective predictor)

^b^ *p* value adjustments (Bonferroni correction) were done for the different vowels within one acoustic parameter and one predictor

^c^Estimates for the predictor gender were gained from the *LMM*, with male speakers included in the intercept. Estimates refer to the female speakers in comparison to the male speakers

^d^Values base on log transformed data

**Table 3:** Results of the post-hoc analyses on the influence of encoding condition on the acoustic structure of vowels

Parameter	Vowel	Encoding condition		Estimate	*SE*	z value	*p* ^a^
Amplitude ratio	a	Authentic	Actor	0.552	0.115	4.949	<.001***
		Authentic	Non-actor	−0.423	0.109	−3.895	<.001***
		Actors	Non-actors	−0.975	0.123	−7.941	<.001***
	e	Authentic	Actor	0.385	0.121	3.177	.004**
		Authentic	Non-actor	−0.323	0.125	−2.591	.029*
		Actors	Non-actors	−0.709	0.133	−5.323	<.001***
Peak frequency	a	Authentic	Actor	0.196^b^	0.077^b^	2.550	.0323
		Authentic	Non-actor	−0.231^b^	0.075^b^	−3.093	.006**
		Actors	Non-actors	−0.428^b^	0.084^b^	−5.077	<.001***
	e	Authentic	Actor	0.103^b^	0.567^b^	1.814	.209
		Authentic	Non-actor	−0.163^b^	0.058^b^	−2.798	.015*
		Actors	Non-actors	−0.266^b^	0.063^b^	−4.243	<.001***
	i	Authentic	Actor	0.022^b^	0.050^b^	0.450	1
		Authentic	Non-actor	−0.206^b^	0.045^b^	−4.555	<.001***
		Actors	Non-actors	−0.229^b^	0.052^b^	−4.381	<.001***
First formant	a	Authentic	Actor	−0.444^b^	0.105^b^	−4.299	<.001***
		Authentic	Non-actor	−0.052^b^	0.102^b^	−0.511	1
		Actors	Non-actors	0.392^b^	0.114^b^	3.442	.002**

Asterisks mark the significance level at *p < .05; **p < .01; ***p < .001

^a^Adjusted *p* values (Bonferroni correction)

^b^Values base on log transformed data

The factor *emotion* influenced the parameters F0 and peak frequency, as well as to a lesser degree the harmonic-to-noise (for vowel “i”) and the amplitude ratios (for vowel “i”). Anger stimuli deviated most strongly from the other emotions by possessing higher F0 and peak frequencies (Fig. 2). Speaker gender influenced the acoustic structure of vowels most strongly (Table 2). Women spoke with a higher F0 and peak frequency, increased bandwidths of the first formant, higher harmonic-to-noise ratio and lower amplitude ratio.

#### Speed of Speech

**Fig. 3:**
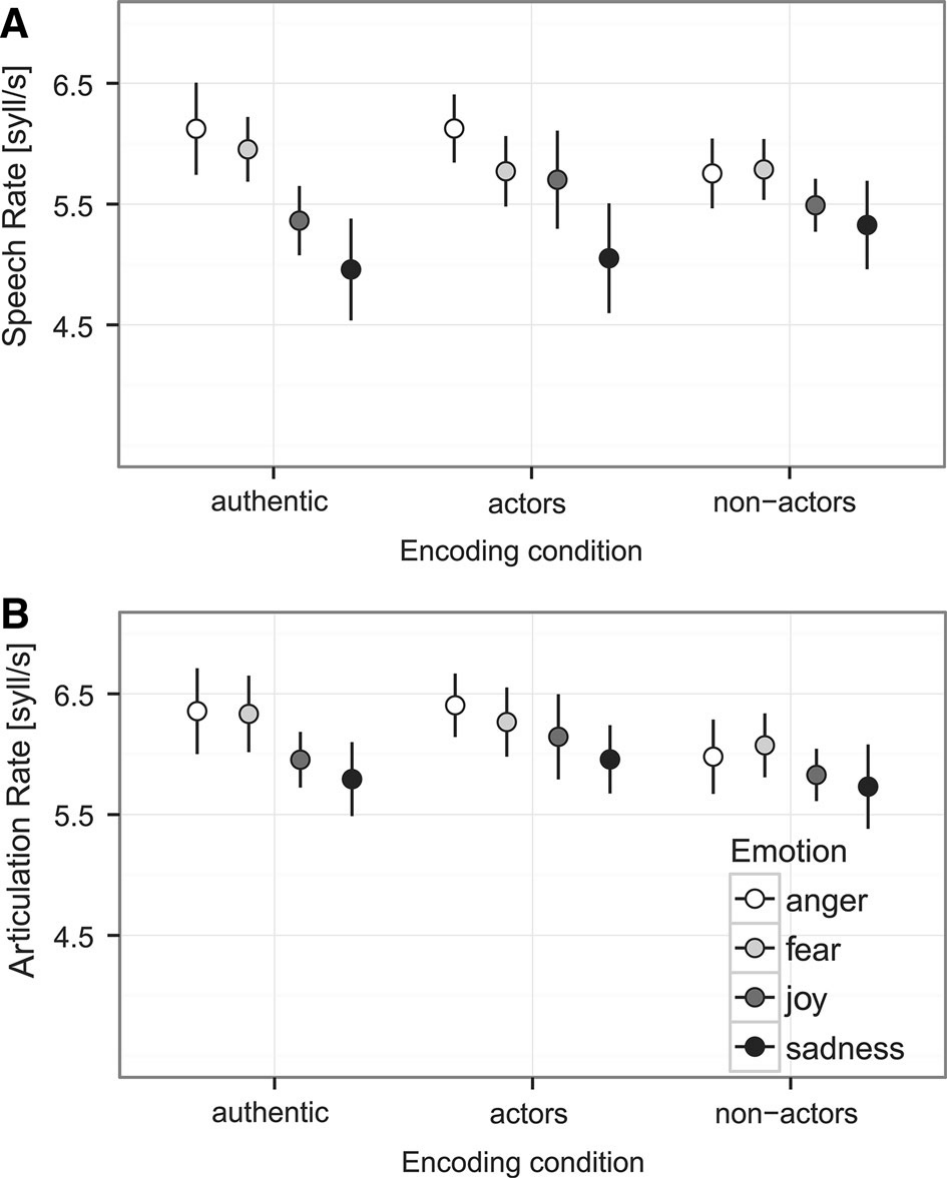
Speech tempo for the different encoding conditions and emotion categories. Mean ± *SEM* is given for **a** speech rate and **b** articulation rate

Speech rate and articulation rate were not affected by the three encoding conditions, the four emotion categories, or their interaction (comparison of full with null model: speech rate *χ* ^*2*^ = 9.2631, *df* = 11, *p* = .598; articulation rate *χ* ^*2*^ = 5.797, *df* = 11, *p* = .887, Fig. 3).

#### Pitch Variability

Pitch variability *(F0*-*SD)* was affected by *encoding condition* (*χ* ^*2*^ = 13.48, *df* = 2, *p* = .001). The post hoc comparison revealed a flatter prosody in authentic speech compared to both acting conditions [authentic—actors: estimates (log transformed data) ± *SE* = −0.3114 ± 0.097, *z* = −2.879, *p* = .012; authentic—non-actors: −0.375 ± 0.109, *z* = −3.45, *p* = .002, non-actors—actors: 0.063 ± 0.116, *z* = 0.547, *p* = 1]. Pitch variability for authentic speech was 23.24 Hz (±1.94 Hz SEM), while actors speech was characterized by a variability of 31.00 Hz (±2.41 Hz SEM), and non-actors speech by 33.07 Hz (±2.36 Hz SEM).

## Discussion

In terms of their acoustic characteristics, vocal expressions of emotions delivered by professional actors were not more similar to authentic expressions than the ones by non-actors. Moreover, vocal expressions by professional actors and non-actors evoked similar recognition patterns. Thus, our findings do not support the hypothesis that compared to non-actors, professional actors have a superior ability to produce emotional portrayals that resemble spontaneous expressions (hypothesis 1). Our results furthermore do not support the view that play-acted expressions in general, and the ones by non-professional actors in particular, are necessarily stereotyped caricatures of authentic expressions. The lack of an interaction between encoding condition and emotion in the acoustic variables we analyzed indicates that emotions were expressed in a similar fashion in all of the three recording types (but see Spackman et al. 2009 for contrasting results).

Nevertheless, we found acoustic differences between the encoding conditions. Acting in general was distinguished from the authentic recordings by a more variable speech melody (see also Audibert et al. 2010; Williams and Stevens 1972). Interestingly, high variability in pitch contour has been related to more aroused emotions, such as anger, while low variability characterize less aroused expressions such as sadness (Juslin and Laukka 2003; Scherer 1986). The different intonation may thus interact with emotion perception, which might explain the differences in emotion recognition for authentic speakers, actors and non-actors. The variable speech melody in play-acted expressions might be confounded with anger perception, affecting the high recognition rates for play-acted anger. Low variability on the other hand might be misinterpreted as sadness, and facilitates the sadness recognition for authentic speech tokens.

As in previous studies, the recognition of encoding condition was rather low (Drolet et al. 2012; Jürgens et al. 2013; Porter and ten Brinke 2009). Listeners were thus unable to reliably recognize whether an expression was acted or not. Expressions by experienced actors were rated as “play-acted” more often than the ones by non-actors, which were not recognized as “play-acted” above chance level. The differences in articulation supports this notion, as acoustic profiles of non-actors’ speech resembled the structure of authentic speech tokens, while the acoustic profiles of professional actors differed from the other two categories. The acoustic differences of vowels between the encoding conditions might be caused by variation in articulation, as a result of speech training, rather than on differences in emotion encoding between acting and spontaneous expression. These results indicate that acting training interacts with the perception of authenticity and supports hypothesis 2 (see also Krahmer and Swerts 2008).

Although listeners appear to process some of the acoustic variation between encoding conditions implicitly (Drolet et al. 2012, 2014), they appeared to be unable to use these cues for recognizing the encoding condition, as evidenced by the poor hit rates. One important open question is the source of variation in speech melody, specifically whether it is due to acting, or to variation between speaking styles, such as reading (Batliner et al. 1995; Eskenazi 1992; Laan 1997). Considering that during the acting conditions the actors and non-actors were not asked to learn the sentences by heart, the differences may also reflect the reading and not the acting process. Future studies will be needed to clarify this issue.

The effect of emotion on the acoustic structure is consistent with the literature (Hammerschmidt and Jürgens 2007; Scherer 2003), although effect sizes (as well as the corresponding recognition rates) for both the acted and spontaneous expressions were weak compared to previous studies on vocal expressions (Hammerschmidt and Jürgens 2007; Laukka et al. 2005; Scherer 2003). Our speech tokens were not preselected and were taken from long text sequences in which not all of the words were equally emotionally accentuated (in contrast to studies in which only one word was expressed emotionally). This procedure seems suitable for avoiding exaggerated portrayals and thus increasing realism of play-acted expressions. The low emotional content may reflect the actual emotionality transmitted via speech (in contrast to emotional outbursts) and emphasized the importance of studying realistic expressions to understand daily communication.

The fact that vocal expressions of emotions are so easily play-acted, without being detected as such, contributes to the discussion about the reliability of emotional expressions. The question is whether emotion expressions need to be tied to specific affective states including subjective feeling or physiological changes. (Dezecache et al. 2013; Fernández-Dols and Crivelli 2013; Mehu and Scherer 2012). For facial expressions, the Duchenne smile (smiling including the zygomaticus major and the orbicularis oculi muscle) was suggested to signal felt happiness only, while smiles without activation of the orbicularis oculi were classified as faked (Ekman et al. 1990). Recent studies, however, demonstrated the common use of Duchenne smiles in acted expressions (Krumhuber and Manstead 2009, for discussion Riediger et al. 2014). As noted above, we did not measure physiological state, or subjective feelings of our spontaneous speakers and do not claim to test coherence between feelings and expressions. However, the general similarity in expression patterns in the three encoding conditions is in keeping with the view that in humans, expressions of emotions can be successfully decoupled from subjective feelings (Fernández-Dols and Crivelli 2013).

As in every study using daily life data, our study does suffer from some limitations, mostly due to the nature of our authentic expressions being recorded by radio reporters. For this reason, we were unable to create fully comparable stimuli. The recording quality of real-life situations may be substantially worse compared to the play-acted ones. We did, however, try to obtain play-acted recordings under a variety of acoustic conditions. If the higher recognition rates for authentic stimuli were simply explained by the recording quality, play-acted expressions would have had much higher recognition rates than they actually did (for other studies finding a bias to preferentially choose authentic see Gosselin et al. 1995; Jürgens et al. 2013; Levine et al. 1999). In any case, our current study aimed to compare play-acted expressions by trained professional actors and non-actors and these recording conditions were equivalent. Another limiting aspect is the fact that our sample of decoders consisted mainly of students, thus being rather homogeneous and limited for a generalization of results. However, in a preceding study, the authentic and professionally acted stimuli were rated by students from three different cultures (Germany, Romania, and Indonesia) (Jürgens et al. 2013). All three study populations showed highly similar rating patterns, suggesting that some of our findings may be generalizable. On the other hand, our sample of speakers was rather heterogeneous, with non-actors being older than the professional actors. Previous studies showed that decoders are generally more accurate in judging expressions from their own age category, while older encoders are supposed to express emotions less distinctly (Borod et al. 2004; Riediger et al. 2011), both suggesting an advantage for the younger encoder group (“actors”). This effect does not appear to be strong in our sample, as both acting conditions were rated similarly, despite the age difference of the speakers. One advantage of our stimulus set is the high number of speakers, which minimizes the probability that our results are based on individual differences between speakers rather than on the group differences. Future studies should focus on individual differences in emotion encoding to disentangle the effects of age, gender, non-professional acting experience, voice use, and even current mood of speakers on the play-acting of emotion, factors that we could not consider in our analysis.

In summary, our study centered on methodological issues that may have strong effects on the interpretation of previous results, and are relevant for the planning of future studies. We showed that compared to professional actors, non-actors are equally capable of transmitting emotional information via the voice when asked to portray an emotion; additionally non-actors’ expressions were perceived as more realistic. For future studies, recordings of daily life emotion expressions should clearly be preferred, but recording spontaneous emotion behavior is unfortunately rarely possible. As an alternative, our findings on vocal expressions speak for the use of non-professional actors when realistic stimulus databases are required.

## Appendix

Examples of transcripts and stimulus texts used for the play-acted recordings (taken from Jürgens et al. 2011, Appendix 1). Only the words in quotation marks were used for the rating study and the acoustic analysis.

### Male Spoken Anger

#### Context

Two fighting dogs attacked 6-year-old V. in the schoolyard. He was bitten to death. Fighting dogs are a big problem in the area and people do not feel protected by the police. They are furious and are looking for a culprit. The anger is directed to the police. The people are shouting at a police officer, blaming him for being too late.

Man:

- Original (German): Der Kiosk ruft vor Viertelstund an, “*nach Viertelstund*“ kommt ihr erst, oder was?”
- Translation: The kiosk called 15 min ago, you only come “*after 15* *min*” or what?

### Female Spoken Sadness

#### Context

The 73-year-old W. was attacked in his shop by two 16-year-old boys. He was robbed and stabbed to death. It is the date of the funeral. A weeping woman reports.

Woman:

- Original (German): “*Ich kenn den 43 Jahr*“. Und er war für uns alle ein Freund. Und ich finde das furchtbar, was da passiert ist.
- Translation: “*I have known him for 43* *years*”. And he was a friend, for all of us. And I think what happened is dreadful.

### Male Spoken Joy

#### Context

The Fall of the Berlin Wall. A citizen of the German Democratic Republic reports excitedly and happily about the border crossing.

Man:

- Original (German): Vorhin haben sie noch einzeln durchgelassen. Dann haben sie das Tor aufgemacht, “*und jetzt konnten wir alle*“ so, wie wir waren, ohne vorzeigen, ohne alles, konnten wir gehen.
- Translation: Previously they let the people pass individually. Then they opened the gate and “*now we could all*”, as we were, without showing anything, without everything, we could go.

### Female Spoken Fear

#### Context

The hundred year flood at the Oder threatens whole villages. The water is rising and an inhabitant of an especially low-lying house reports her fears.

Woman:

- Original (German): Grade unser Haus liegt ziemlich tief. Also 1947 stand das Wasser da schon “*bis zum Fensterkreuz*“. Und wenn das noch schlimmer werden sollte, schätz ich, dass das Haus bald gar nicht mehr zu sehen ist im Wasser. Ja, ich hab ganz dolle Angst
- Translation: Especially our house lies pretty low. Well, 1947 the water was already “*up to the window crossbar*”. And if it should get worse, I guess, that the house won’t be visible anymore in the water. Yes, I am very much afraid.
